# Effects of sleep deprivation on central auditory processing

**DOI:** 10.1186/1471-2202-13-83

**Published:** 2012-07-23

**Authors:** Paulo Breno Noronha Liberalesso, Karlin Fabianne Klagenberg D’Andrea, Mara L Cordeiro, Bianca Simone Zeigelboim, Jair Mendes Marques, Ari Leon Jurkiewicz

**Affiliations:** 1 University Tuiuti of Paraná, Otoneurology Research Center, Curitiba, Brazil; 2 Department of Neuropediatrics, Little Prince Children’s Hospital, Curitiba, Brazil; 3 Department of Neuropsychopharmacology, Pelé Little Prince Research Institute, Curitiba, Brazil; 4 Faculdades Little Prince, Curitiba, Brazil; 5 Department of Psychiatry and Biobehavioral Sciences of the David Geffen School of Medicine, Semel Institute for Neuroscience and Human Behavior, University of California, Los Angeles, USA

**Keywords:** Central auditory processing, Sleep, RGDT, SSWT

## Abstract

**Background:**

Sleep deprivation is extremely common in contemporary society, and is considered to be a frequent cause of behavioral disorders, mood, alertness, and cognitive performance. Although the impacts of sleep deprivation have been studied extensively in various experimental paradigms, very few studies have addressed the impact of sleep deprivation on central auditory processing (CAP). Therefore, we examined the impact of sleep deprivation on CAP, for which there is sparse information. In the present study, thirty healthy adult volunteers (17 females and 13 males, aged 30.75 ± 7.14 years) were subjected to a pure tone audiometry test, a speech recognition threshold test, a speech recognition task, the Staggered Spondaic Word Test (SSWT), and the Random Gap Detection Test (RGDT). Baseline (BSL) performance was compared to performance after 24 hours of being sleep deprived (24hSD) using the Student’s *t* test.

**Results:**

Mean RGDT score was elevated in the 24hSD condition (8.0 ± 2.9 ms) relative to the BSL condition for the whole cohort (6.4 ± 2.8 ms; p = 0.0005), for males (p = 0.0066), and for females (p = 0.0208). Sleep deprivation reduced SSWT scores for the whole cohort in both ears [(right: BSL, 98.4 % ± 1.8 % vs. SD, 94.2 % ± 6.3 %. p = 0.0005)(left: BSL, 96.7 % ± 3.1 % vs. SD, 92.1 % ± 6.1 %, p < 0.0001)]. These effects were evident within both gender subgroups [(right: males, p = 0.0080; females, p = 0.0143)(left: males, p = 0.0076; females: p = 0.0010).

**Conclusion:**

Sleep deprivation impairs RGDT and SSWT performance. These findings confirm that sleep deprivation has central effects that may impair performance in other areas of life.

## Background

Sleep is a reversible state of consciousness characterized by the temporary suspension of perceptual/sensory phenomena and voluntary motor activity that naturally occurs at regular intervals, alternating with wakefulness. Although the precise biological functions of sleep have yet to be resolved, it is clear that sleep is vital to mammalian survival [1]. Furthermore, sleep plays an important role in learning and memory consolidation [2,3]. Inadequate sleep is a growing problem worldwide that affects healthy individuals as well as people with various disorders [4,5]. The lifestyles of modern societies are increasingly associated with stress and, consequently, poor sleep quality. Furthermore, reductions in the total hours of sleep obtained have tremendous impacts on various bodily systems. Reduction in hours slept has been shown to have negative effects on the immune, metabolic, and endocrine systems [6]. For example, 24.5 hours of sleep deprivation was recently shown to increase blood pressure in healthy normotensive adults [7]. And, although the etiology of mood and behavioral disorders is complex and not yet fully understood, a clear link between sleep deprivation and psychiatric conditions has been established [8,9].

Although the impact of sleep deprivation has been studied extensively in various experimental paradigms from basic animal research, such as the effects of SD on hippocampal neurogenesis [10], to sophisticated human imaging studies [11], there have been very few studies that have addressed the impact of sleep deprivation on central auditory processing (CAP). CAP can be understood as a set of neurophysiological and neurochemical mechanisms that occur in the auditory system in response to acoustic stimuli. Cognitively, CAP is critical for language comprehension and is responsible for sound localization and lateralization, sound discrimination, auditory pattern recognition, and the temporal aspects of hearing (including auditory masking, resolution, integration, and ordering), as well as for the ability to negotiate competing or degraded acoustic signals [12].

Central auditory processing disorder (CAPD) is characterized by listening difficulties despite a normal audiogram. Sleep deprivation impacts several functions associated with prefrontal cortex (PFC) activity [13,14] and auditory temporal resolution is a perceptual function associated with PFC activity [15]. Thus, it is reasonable to posit that sleep deprivation may impair CAP through circuits involving the PFC.

The aim of the present study was to evaluate the impact of sleep deprivation for a period of 24 hours on CAP in neurologically and psychologically healthy adults. CAP in a baseline control state and a 24-hour sleep-deprived state (24hSD) was assessed using the Random Gap Detection Test (RGDT) and the Staggered Spondaic Word Test (SSWT). The RGDT measures temporal resolution processing (auditory timing) abilities in the auditory domain. The SSWT measures recognition of overlapping sounds presented to the auditory central nervous system dichotically [16]. Although the effects of sleep deprivation have been extensively studied in several paradigms, including sophisticated neuropsychological and imaging studies [11,17,18], there are still many questions to be answered with respect to how brain circuits are integrated in healthy and adverse conditions. With the objective of contributing to the latter, we designed the present investigation to test the hypothesis that 24 hours of sleep deprivation would negatively affect CAP in otherwise healthy adults.

## Methods

### Participants

Inclusion criteria included the following: 18–40 years of age; physically and psychologically healthy, and good sleep habits. The exclusion criteria were: diagnosis of sleep disorders, hearing loss, or other neurological diseases; family history of hereditary diseases, as assessed by physical examination and history; and medication use within the 7 days preceding the initial hearing evaluation and baseline (BSL) CAP examination. Use of a medication during the period between the BSL and 24hSD test sessions would also result in exclusion from the study.

A total of 30 healthy volunteers met the inclusion criteria and agreed to participate in the study. The cohort included 17 females (56.7 %) and 13 males (43.3 %), with a mean ± standard deviation, age 30.7 ± 7.1 years. All participants indicated that they did not smoke, consume any medications, stimulants, caffeine, or alcohol for at least 24 hours prior to the BLS and 24hSD test sessions. We obtained informed formal written consent from all participating subjects, and the research project was approved by the Ethics Committee on Research Involving Human Subjects (registration number CEP 0527/08 at the Hospital Pequeno Principe, Curitiba, Brazil).

### Study design

An initial hearing evaluation consisted of pure tone air (250–8000 Hz) and bone (500–4000 Hz) conduction threshold audiometry, a speech recognition threshold test, a speech recognition percent index, and acoustic impedance measurements. Preliminary audiometric evaluations included tympanometric curve measurements for which a type A tympanometric (tympanogram) finding (i.e., maximum impedance at or near 0 Pa) indicated normal middle ear physiology and a type Ad tympanometric curve (i.e., an extremely high impedance peak) was taken as evidence of disjunction of the ossicular chain and/or abnormalities in the tympanic membrane.

CAP was assessed by the RGDT and the SSWT at two different times (BSL and 24hSD) with an inter-test interval of 2–3 months, during which the participants were allowed to continue to consume alcoholic or caffeinated beverages. The BSL CAP tests were conducted immediately after the initial hearing evaluation. The RGDT and the SSWT were conducted using a D88 discman, coupled to a Madsen Itera II diagnostic audiometer (GN Otometrics, Tasstrup, Denmark) with TDH39 headphones.

The first CAP session, BSL, was performed in a normal (no sleep deprivation) control condition and the second test was conducted in the 24hSD state following 24 hours of no sleep. In consideration of the participants’ convenience and safety, SD was carried out on Fridays, and the 24hSD measurements were conducted on Saturdays. A research assistant remained with the subjects throughout the night to ensure that they did not fall asleep during the experimental sleep deprivation period. Most of subjects chose to watch TV during the sleep deprivation period. All testing was performed at the phonoaudiology clinic of the University of Tuiuti in Brazil.

### RGDT

The RGDT, which is described in detail elsewhere [19,20], produces time scores wherein a lesser time (lower score) indicates better performance. In our RGDT protocol, pairs of pure tones were presented in the following frequencies: 500 Hz, 1000 Hz, 2000 Hz, and 4000 Hz. The procedure was repeated for each frequency, resulting in a trial that included four single-frequency runs. The intervals between paired tones ranged from 0 ms to 40 ms (specifically, 0, 2, 5, 10, 15, 20, 25, 30, or 40 ms), with the order of the intervals being random. The interval between the consecutive presentations of tone pairs was 4.5 seconds to allow an adequate response time for the participants.

The RGDT was scored for the smallest interval between paired tones for which the individual was able to identify the presence of two discrete stimuli. This identification assessment involved an assessment of gap detection, which reflects the ability to resolve time differences. Within three noise bursts (two of which were unbroken), the subject was asked to identify which one included a silent interval ("gap"). Each person responded to the stimuli verbally or by holding up one or two fingers to indicate whether s/he heard one or two tones. Normal gap detection threshold was considered to be 2–20 ms. A gap detection threshold >20 ms was considered abnormal.

### SSWT

The SSWT, also described in detail elsewhere [16], produces percentage scores wherein a greater percentage (higher score) indicates better performance. The participants were presented with spondaic words (words with two syllables of equal stress) in an overlapping fashion such the second syllable of the first spondaic word occurred at the same time as the first syllable of the second spondaic word. One ear (the leading ear) was presented with the first syllable of the first spondaic word in isolation (non-competing), followed by the second syllable of the first spondaic word in a dichotic mode (competing). The other ear (the lagging ear) was presented with the second spondaic word such that the first syllable was presented in dichotic mode (during presentation of the second syllable of the first spondaic word to the leading ear) and the second syllable presented in isolation. Each ear served as the leading ear for half of the test presentations. The stimuli were presented at an intensity of 50 dB (SL re:RT/PTA). The testing time was 8 minutes. Error scores were calculated for each ear in both competing and non-competing modes.

### Statistical analysis

Two-tailed paired student’s *t* test was used to compare data between the BSL and 24hSD conditions. Two-way analyses of variance (ANOVAs) were conducted to test whether there were any significant differences among the sexes. P < 0.05 was considered statistically significant. All data are reported as means ± SDs (standard deviations).

## Results

### Hearing ability of participants

Preliminary audiometric evaluations using tympanometric curve measurements confirmed that all 30 participants had normal hearing. Most of the participants, 28/30 (93.4 %), were found to have type A tympanometric curves bilaterally (maximum impedance at or near 0 Pa); the remaining 2 (6.7 %) had type Ad tympanometric curves (with an extremely high impedance peak in the curve). Additionally, we found that 3/30 subjects (10.0 %) had a bilateral absence of the ipsilateral reflex, 1 (3.4 %) had an absence of the contralateral reflex on the right only, and 1 (3.4 %) had an absence in all frequencies tested. Most of the volunteers, 25/30 (83.4 %), had acoustic reflexes at all frequencies tested.

### SD worsens RGDT performance

**Figure 1:**
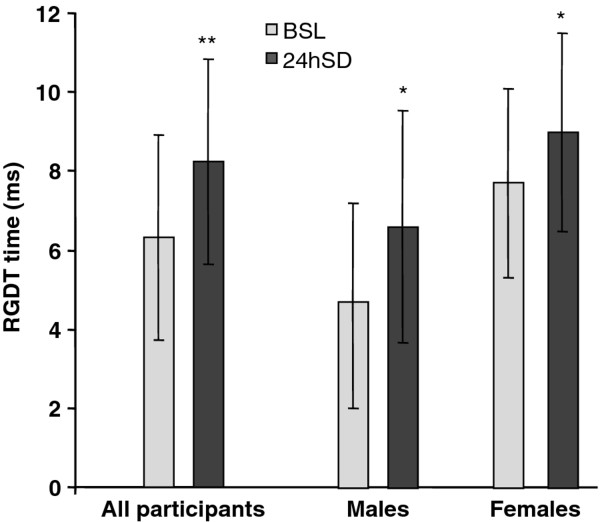
**RGDT performance was impaired by sleep deprivation.** Mean RGDT times following 24 hours of sleep deprivation (24hSD) were significantly greater than those observed during the baseline condition (BSL) without sleep deprivation. Error bars show standard deviations. *p ≤0.01, **p ≤0.001 vs. BSL for whole study cohort (left).

As shown in Figure 1, the mean RGDT score for the entire group in the non-sleep deprived BSL condition was 6.4 ± 2.8 ms (range, 2.0–13.7 ms), and was elevated to 8.0 ± 2.9 ms (range, 2.7–13.7 ms) in the 24hSD condition (*p* = 0.0005). Subsequent analysis of the RGDT data by gender subgroup showed that this effect was not gender dependent, as this pattern of results was replicated within both the male and the female subgroups. The mean RGDT score for males increased from 4.7 ± 2.7 ms (range, 2.0–10.0 ms) in the BSL condition to 6.6 ms ± 2.9 ms (range, 2.7–10.0 ms) in the 24hSD condition (*p* = 0.0066). Likewise, the mean RGDT score for females increased from 7.7 ± 2.4 ms (range, 5.0–13.7 ms) in the BSL condition to 9.0 ± 2.5 ms (range, 5.0–13.7 ms) in the 24hSD condition (*p* = 0.0208). Although both sex subgroups exhibited the aforementioned increases from BSL to the 24hSD condition, an ANOVA showed that males and females differed from each other at BSL (p = 0.0026) and in the 24hSD condition (p = 0.0206).

### SD worsens SSWT performance

**Figure 2:**
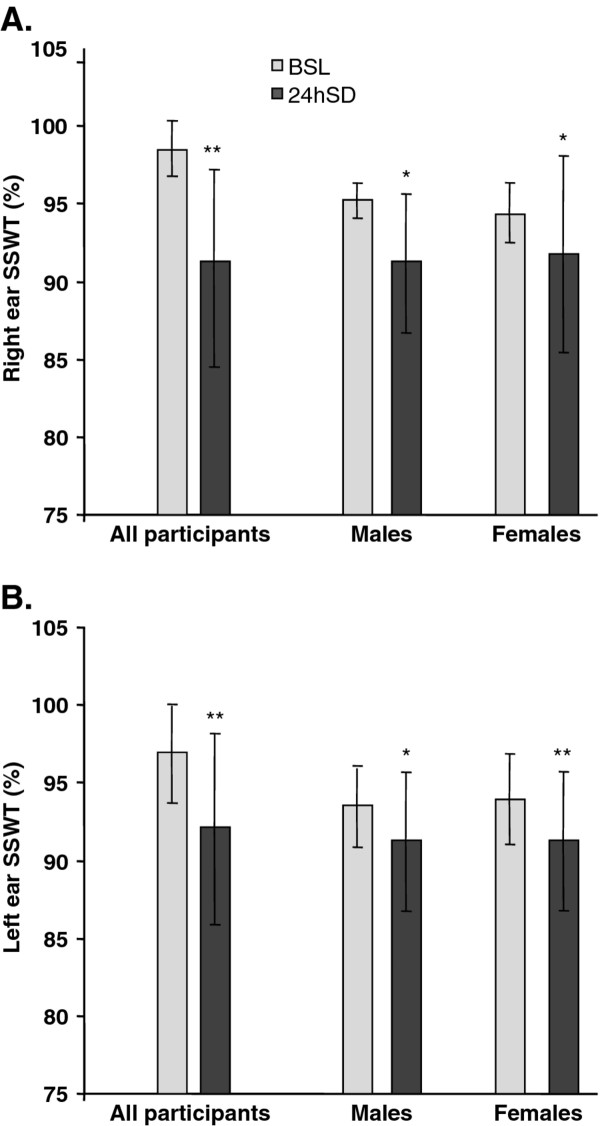
**SSWT performance was impaired by sleep deprivation.** Mean SSWT times following 24 hours of sleep deprivation (24hSD) were significantly reduced compared to those observed during the baseline condition (BSL) without sleep deprivation. Error bars show standard deviations. *p ≤ 0.01, **p ≤ 0.001 vs. BSL for whole study cohort (left) or within gender subgroups.

As illustrated in Figure 2a and b, the study cohort as a whole showed significant worsening of performance in the SSWT in both the right ear and the left ear following sleep deprivation. In the right ear, the group mean fell from 98.4 ± 1.8 % (range, 92.0–100.0 %) in the BSL condition to 94.2 ± 6.3 % (range, 80.0–100.0 %) in the 24hSD condition (*p* = 0.0005). In the left ear, the group mean fell from 96.7 ± 3.1 % (range, 90.0–100.0 %) in the BSL condition to 92.1 ± 6.1 % (range, 80.0–100.0 %) in the 24hSD condition (*p* < 0.0001).

An ANOVA of the SSWT data by gender subgroup showed that the 24hSD effect on SSWT performance was not gender dependent (Fig. 2a and b). In the right ear of males, the mean SSWT score fell from 98.5 ± 1.3 % (range, 97.5–100.0 %) in the BSL condition to 93.9 ± 5.2 % (range, 85.0–100.0 %) in the 24hSD condition (*p* = 0.0080), and in the left ear of males, the mean SSWT score fell from 96.5 ± 2.9 % (range, 92.5–100.0 %) in the BSL condition to 91.5 ± 6.9 % (range, 80.0–100.0 %) in the 24hSD condition (*p* = 0.0076). Similarly, in the right ear of females, the mean SSWT score fell from 98.4 ± 2.2 % (range, 92.0–100.0 %) in the BSL condition to 94.4 ± 7.3 % (range, 80.0–100.0 %) in the 24hSD (*p* = 0.0143), while in the left ear of females, the mean SSWT score fell from 96.9 ± 3.4 % (range, 90.0–100.0 %) in the BSL condition to 92.5 ± 3.4 % (range, 80.0–100.0 %) in the 24hSD condition (*p* = 0.0010). There were no gender differences in SSWT results at BSL or in the 24hSD condition (p > 0.05).

## Discussion

The overall aim of this study was to investigate the impact of sleep deprivation on CAP in a group of healthy adults by comparing performance in the RGDT and the SSWT in a BSL (rested) condition versus after 24 hours of sleep deprivation. We showed that this relatively short period of sleep deprivation significantly impaired CAP in both males and females. More specifically, the increase in the average time needed to identify the interval of silence between paired sound stimuli in the RGDT that was observed following sleep deprivation demonstrated a negative impact of sleep deprivation on temporal resolution ability. Additionally, the decrease in SSWT performance observed in the 24hSD condition relative to the BSL condition indicates that 24 hours of sleep deprivation impaired the participants’ ability to transfer auditory information between the cerebral hemispheres via the posterior portions of the corpus callosum [21]. Although there were differences between males and females at BLS and at the 24hSD in the RGDT, the overall CAP impairments in male and female subjects followed a similar pattern, indicating that the effects of sleep deprivation on CAP are not related to gender.

Acute sleep deprivation has a major impact on brain neurophysiology and can trigger changes in cortical and subcortical structures. Notably, prolonged wakefulness can alter frontal lobe functioning, affecting language expression and perception, reducing one’s ability to perform creative processes, pay attention, and concentrate [22]. The neurophysiological impairment of the lateral (neocortical) temporal lobe after acute sleep deprivation is manifested in changes of auditory abilities, including sound perception and the processing of auditory information [23]. On the other hand, sleep deprivation-associated neurophysiological changes in the medial temporal lobe structures, especially the hippocampal formation and amygdala, may impair memory (e.g., the ability to remember words) and may alter mood and behavior [22,24]. Sleep deprivation can also impair functioning of the posterior temporal and parietal regions, resulting in a decreased ability to identify the main focus of attention [25].

CAP functions are heavily dependent on alertness and concentration. Thus, acute sleep deprivation that temporarily affects attention could, consequently, also alter the phenomena that constitute the neurobiological bases of CAP. From a neuropsychological perspective— which predicts that all memory-based tasks should be affected by SD [13]—studies have shown that language tasks that require sustained attention and higher level processing, such as reading comprehension, were negatively affected by sleep deprivation, whereas other tasks that rely on basic language processing, such as order recall or category recall, were not affected [26]. Furthermore, some executive functions, the ability to execute complex tasks (i.e., creative problem solving), decision making, attention, and vigilance have been shown to be impaired by sleep loss [27,28]. However, other researchers have found that acute sleep deprivation did not impair performance in complex cognitive tasks that require critical thinking and reasoning [26].

The aforementioned findings [26] may seem paradoxical since the prefrontal cortex (PFC) is activated during attention, vigilance, and complex cognitive tasks. The fact that studies have shown that some cognitive tasks that could be considered stimulating and that require active and frequent responses were less affected by acute sleep deprivation [26] could be interpreted as indicating that sleep deprivation-resistant cognitive tasks, regardless of complexity or stimulation, could be activating the reward dopaminergic system, and thus over-running the impairing effects of acute sleep deprivation. For instance, using functional magnetic resonance imaging, Libedinsky and colleagues [29] showed that when subjects under total sleep deprivation were presented with images that produced anticipation of monetary or social rewards, the ventromedial PFC was activated. The mesocorticolimbic brain reward circuit involves activation of the dopaminergic system, similar to a pharmacological stimulant. Such increases in dopamine (DA) release, especially in the PFC, could counteract some of the cognitive deficits associated with sleep deprivation [30].

The neurochemical mechanisms responsible for decreased performance in neurocognitive and attention tests after varying periods of sleep deprivation are not fully understood. However, some studies have suggested that DA may be responsible for the behavioral changes observed following sleep deprivation. Studies in rats have shown that brain levels of DA and noradrenaline are increased after 24 hours of sleep deprivation, peaking at 96 hours of sleep deprivation and recovering to normal levels after 24 hours of rest [31]. Imaging studies in humans have shown that DA release is significantly increased after 24 hours of total sleep deprivation [11]. Other studies have shown that, although the dopamine transporter (DAT) was not influenced by 4 nights of REM sleep deprivation and 2 nights of total sleep deprivation, the sleep deprived participants showed an increase in DAT density and it was positively correlated with estradiol concentration [32].

While, the implications of the above DA system findings have yet to be elucidated, we do know that DA plays a major role in attentional networks [33]. The ability to maintain attention for long periods of time has been linked to DA actions in the anterior frontal cortex [33]. The neural network associated with attention appears to involve the integrated operation of the prefrontal, temporal, and parietal cortices, the basal ganglia, and the cerebellum [34,35]. Topographically, the cortical regions associated with attentional focus partially overlap with the auditory association cortex. Thus, theoretically, it is possible that the attentional network could directly influence the ability to process auditory information. Indeed, recent research has shown that the neurophysiology of the frontal, temporal, and parietal lobes appears to be severely impaired after 24 hours of sleep deprivation; such impairments may consequently impair CAP [36].

Memories are essential to accurate processing of auditory information [37,38]. Although memory consolidation is a complex phenomena, a convergence of evidence suggests that sleep may play a critical role in memory consolidation [3]. Indeed, people commonly show impaired memory following acute sleep deprivation [3]. Vecsey and colleagues [39] documented decreased levels of adenosine 3,5-cyclic monophosphate (cAMP) in the hippocampal cells of sleep deprived rats; cAMP is a major second messenger involved in diverse biological phenomena, including memory consolidation [39].

We chose to use the RGDT in this study because auditory temporal resolution represents the neurophysiological basis of CAP. Temporal resolution allows us to understand human speech continuously. Thus, impairment of this ability may make it impossible for one to recognize discrete, meaningful variations in speech. Indeed, virtually all auditory information is influenced by the variable of time [40].

The literature is inconsistent with respect to how much sleep deprivation is needed to produce effects on verbal and executive cognitive processes. Some authors have suggested that sleep deprivation less than 40 hours in duration may not significantly affect complex cognitive processes [41,42], whereas other authors have reported findings of changes in mood, behavior, and attention after only 24 hours of continuous wakefulness [18]. Moreover, differential responses to sleep deprivation across different individuals may depend on several endogenous and exogenous factors, such as the period without sleep, circadian rhythms, and personal motivation, as well as behavioral and personality characteristics [1].

Our findings should be considered in light of some limitations. Firstly, we did not ask the participants to wear an activity meter (actimeter) or to complete a sleep log prior to testing. It should be noted however that sleep logs provide only subjective information of the participants’ sleep habits. Moreover, since all of the participants were professionals with a college degree, and were willing to participate in study without any financial compensation, we assumed that they were honest and serious about our study.

Secondly, we did not explore in greater detail the gender differences observed in the RGDT condition. The main objective of the present study was not to investigate the presence of gender related differences. Nonetheless, some studies have documented gender related differences in cognitive performance and in visual and auditory processing of verbal and figural tasks; and sex differences in brain structure in auditory and cingulated regions have been reported [15,43-45]. Certainly, the gender factor could be further studied in a more controlled experiment with a larger number of participants.

Thirdly, since our sample was relatively small, we did not properly counterbalance for order. Ideally, half of the participants could have completed the BSL condition first and the 24hSD second, while the other half could have done the reverse. Finally, we did not collect any type of psychomotor vigilance data, reaction time data, or attentional measurements. Several studies have suggested that sleep deprivation has a major impact on sustained attention [46,47]. Thus, it could be that the observed sleep deprivation-related impairments in RGDT and SSWT performance were a result of brief attentional lapses rather than an impairment of CAP per se. Nevertheless, the results presented set the stage for further investigations and replications under more stringent experimental conditions.

## Conclusion

In conclusion, the results presented here provide a demonstration that acute sleep deprivation for a period of 24 hours is sufficient to significantly worsen CAP in neurologically and psychologically healthy men and women. Impairment of CAP following sleep deprivation may be due the effects of extended wakefulness on neurobiological functions that are critical for the processing of auditory information, including memory, attention, concentration, reaction time, stimulus perception, and behavioral regulation.

## Abbreviations

ANOVA, Analysis of variance; BSL, Baseline; CAP, Central auditory processing; CAPD, Central auditory processing Disorder; DA, Dopamine; DAT, Dopamine transporter; REM, Rapid eye movement; RGDT, Random gap detection test; SD, Standard deviation; SSWT, Staggered spondaic word test.

## Competing interests

The authors declare that they have no competing interests.

## Authors’ contributions

PBNL and ALJ were co-principal investigators of the study. PBNL, ALJ, and BSZ designed the study protocol. PBNL and KFKA collected the data. PBNL, BSZ, MLC, JMM, and ALJ analyzed and interpreted the findings and wrote the manuscript. MLC performed a critical revision of the manuscript for important intellectual content and was responsible for submitting the final approved manuscript. JMM were responsible for the statistical analysis. All authors read and approved the final manuscript.
